# Differential metallothionein expression in oral lichen planus and amalgam-associated oral lichenoid lesions

**DOI:** 10.4317/medoral.22144

**Published:** 2018-04-24

**Authors:** Gabriela-Geraldo Mendes, João-Paulo-Silva Servato, Fabiana-Custódio Borges, Roberta-Rezende Rosa, Carla-Silva Siqueira, Paulo-Rogério de Faria, Adriano-Mota Loyola, Sérgio-Vitorino Cardoso

**Affiliations:** 1 Oral Pathology Area, School of Dentistry, Federal University of Uberlândia (UFU), Uberlândia, Minas Gerais, Brazil; 2 Biopathology Area, School of Dentistry, University of Uberaba (UNIUBE), Uberaba, Minas Gerais, Brazil; 3 School of Medicine, Federal University of Goiás (UFG), Jataí, Goiás, Brazil; 4 Area of Morphology, Biomedical Sciences Institute, Federal University of Uberlândia (UFU), Uberlândia, Minas Gerais, Brazil

## Abstract

**Background:**

Oral lichen planus (OLP) is a chronic inflammatory disease mediated by T cells, which manifests as reticular (white) or erosive (red) lesions, that are eventually painful. Oral lichenoid lesion (OLL) are distinguished from OLP by the presence of precipitating factors. The aim of this study was to evaluate whether the presence of metallothionein, which is involved in anti-apoptotic pathways and the anti-oxidative response, could serve as a differential diagnostic for OLP and OLL.

**Material and Methods:**

We evaluated the expression of metallothionein in 40 cases of OLP and 20 cases of OLL using immunohistochemistry.

**Results and Conclusions:**

White OLP has higher concentrations of metallothionein than red OLP in basal and parabasal layers. Moreover, metallothionein was more frequently observed in the cytoplasm and nuclei of basal cells in OLP patients compared to the same regions of OLL cases. Metallothionein levels are related to OLP severity and may contribute to a differential diagnosis between OLP and OLL.

** Key words:**Oral lichen planus, oral lichenoid lesions, autoimmune disorders, metallothionein, immunohistochemistry.

## Introduction

Oral lichen planus (OLP) is a chronic autoimmune disease of the oral mucosa, mediated by T lymphocytes (1-4). It is found in up to 2% of adults, more frequently in women, in the fifth to sixth decades of life (1-4). Typically, OLP manifests bilaterally in tongue and/or buccal mucosa, as white striae or plaques with striated borders. Some cases occur as atrophic or ulcerated lesions, intermingled with white striae and plaques. Rare forms are associated with formation of bullae. According to clinical presentation, OLP has been classified into white (also described as keratotic or reticular/plaque like forms) or red lesions (also described as atrophic/erosive) (1-4). White forms are usually asymptomatic, but red lesions often result in pain or a burning sensation with significant discomfort to the patient (1-4).

Histologically, OLP is characterized by the presence of a distinct band-like zone of lymphocytic infiltration that is confined to the superficial (juxta-epithelial) aspect of the connective tissue, accompanied by epithelial alterations such as degeneration, necrosis, and apoptosis of basal cells (1-4). In the white form of OLP, the overlying epithelium responds to immunological aggression with hyperkeratosis, while atrophy or ulceration results in the red lesions. Epithelial dysplasia is not expected and, in fact, it has been advocated that lesions with histopathological features of OLP that present epithelial dysplasia should be considered as pathological entities distinct from OLP (1-4).

Oral lichenoid lesion (OLL) is another common immune-mediated disease of the oral mucosa (1-4). It shares the clinical presentation of OLP, with striae, plaques, and eventual epithelial atrophy or ulcer (1-4). In most cases, there is an exposition to a precipitating factor such as medications or dental materials. Single or unilateral lesions, and deep perivascular or mixed infiltrate of inflammatory cells are also typical, but not always present, in OLL cases (1-4). The distinction between OLP and OLL is often a dilemma for clinicians and pathologists. Besides, the precise etiopathogenesis of OLP and OLL are still unknown.

Metallothionein (MT) is a family of four non-enzymatic proteins that provide an intracellular reservoir for zinc, protect cells from toxic heavy metals and oxidative stress, and participate in the regulation of cellular proliferation and differentiation (5-9). Also, MT influences the immune response, probably with chemotactic or regulatory/suppressive roles (5-10).

Allon *et al.* described MT expression in the lowest layers of the oral epithelium and in the inflammatory infiltrate of OLP-affected patients (11). Higher MT expression has been observed in hyperkeratotic (white) rather than in atrophic/erosive (red) lesions of OLP, presumably due to the more pronounced anti-apoptotic response in reticular forms of the disease (11). In contrast, amalgam, which is one of the most frequent precipitating factors for OLL, can stimulate MT expression (12). However, no other previous study has investigated the expression of this protein in OLL, or directly compared MT levels in OLP and OLL. Taken into account these paucities, the aim of this study was to explore the immunohistochemical reactivity for MT in OLP and OLL, to improve our understanding of the pathogenesis of these diseases and to determine whether MT can serve as a differential diagnostic between OLP and OLL.

## Material and Methods

-Patients

This study was approved by the Institutional Committee for Ethics on Research. Forty cases of OLP and twenty OLL associated to amalgam dental fillings were reviewed following Van der Meij and Van der Waal diagnostic criteria (4). Furthermore, cases of OLP were then segregated into two distinct groups according to the clinical presentation of the disease as proposed by Ismail *et al.* (3): reticular and hyperplastic forms, were called white OLP, n=24; and atrophic and erosive forms, were called red OLP, n=16. Data concerning age, gender, ethnicity, primary site, number of lesions, symptoms and symptom duration was retrieved from dental files.

-Immunohistochemical data

Immunohistochemical assays were performed on 3mm thick tissue sections using the streptavidin-biotin-peroxidase method according to standard protocols. After deparaffinization and hydration, sections were subjected to antigen retrieval using EDTA+Tween 20 buffer (pH=8.0) in a decloaking chamber (Biocare Medical, Concord, CA, USA) for 15 minutes at 110°C. Endogenous avidin and biotin binding activities were blocked using skim milk and white eggs. Endogenous peroxidase activity was blocked using three washes of 3% H2O2, each 10 minutes in duration. Incubation with a protein block solution for 15 minutes at room temperature was performed to prevent nonspecific binding (Starr Trek Universal HRP Detection System, Biocare Medical). The sections were incubated with monoclonal mouse anti-metallothionein antibody (clone E9, Dako North America Inc., Carpinteria, CA, USA) at a dilution of 1:400, in a humid chamber at 25°C for 2 hours. Signal amplification and staining were developed using the Starr Trek Universal HRP Detection System (Biocare Medical), and then the sections were counterstained with Harris hematoxylin. As a negative control, primary antibodies were replaced with phosphate-buffered saline. Samples of hepatocellular carcinomas were used as positive controls, controls based on manufacturer’s guidelines.

-Immunostaining evaluation

Evaluation of the immunohistochemical staining was performed with an optical microscope by two investigators (G.G.M. and C.S.S.) using the Quickscore method (13). Briefly, for each epithelial layer of each case the proportion of stained (positive) epithelial cells throughout the section was scored from one to six (1: 0-4%; 2: 5%-19%; 3: 20%-39%; 4: 40%-59%; 5: 60%-79%; 6: 80%-100%). The intensity of staining was also scored from zero to three, corresponding to the presence of negative, weak, intermediate, or strong staining, respectively. The product of scores attributed to proportion and intensity of staining was the final quickscore. Quickscore were given only to distinctive areas of each sub-group. For example, in the white-OLP group only keratinized and acanthotic epithelial areas were analyzed. And, for the red-OLP, just atrophic epitheliums were examined.

-Statistical analysis

Median quickscore values between OLP and OLL, as well as between white and red OLP samples were compared with the Mann-Whitney U test. *P* values < 0.05 were considered significant. Statistical analysis was performed using GraphPad Prism 5 software (GraphPad Software, version 5.01, San Diego, CA).

## Results

-Clinic-pathological data

**Figure 1:**
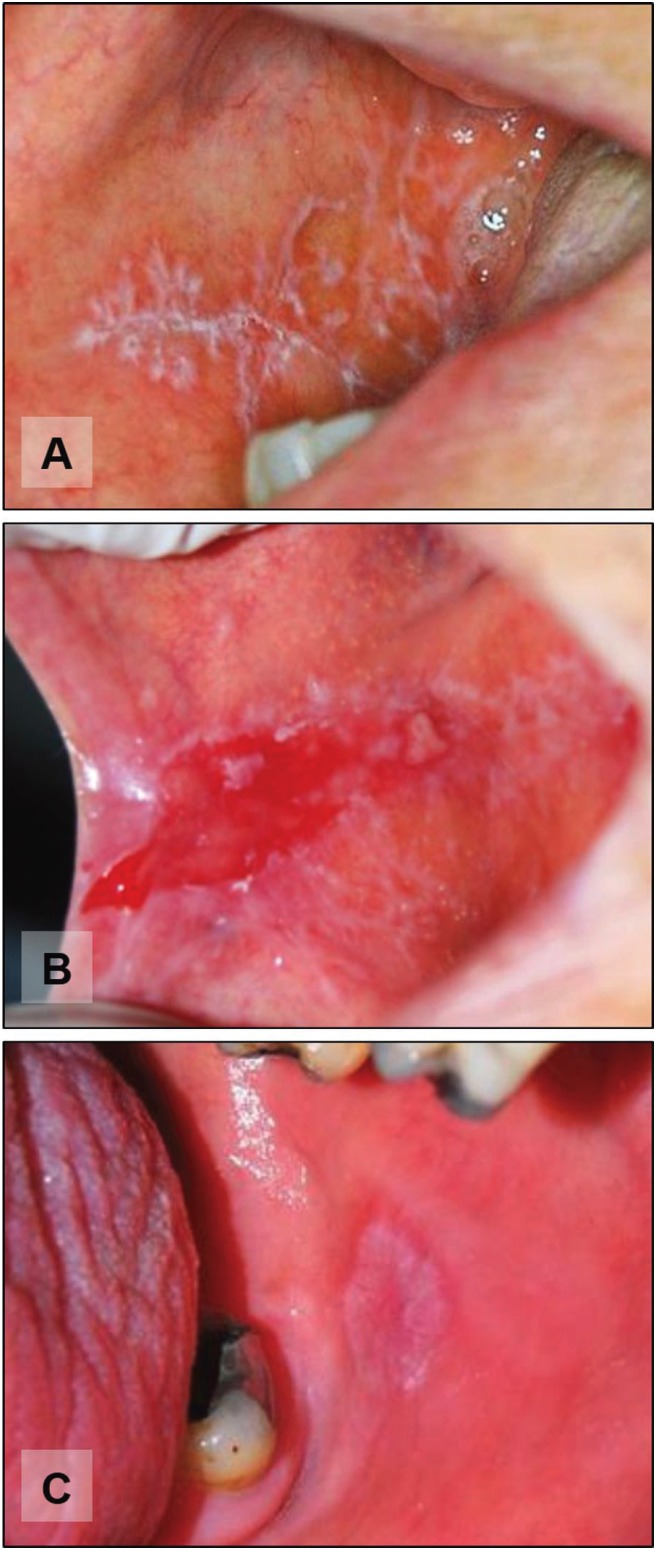
Common clinical aspects observed in the white (A) and red (B) forms of oral lichen planus, as well as in oral lichenoid lesions (C).

As shown in  Table 1, most of the patients were white women, in their fifth decade of life. Buccal mucosa was the most frequent site of involvement. All cases of white OLP was asymptomatic, while pain or burning were reported for all cases of red OLP and in 40% of OLL. Most of OLL lesions were clinically described as white lesions, only four OLL were described as red-white mixed lesions. Representative pictures of the clinical aspect of these lesions are shown in Figure 1.

**Table 1:**
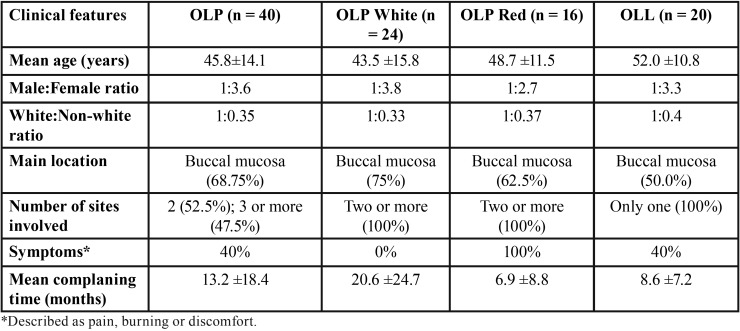
Clinical features of the patients enrolled in the study.

-Immunohistochemical data

**Figure 2:**
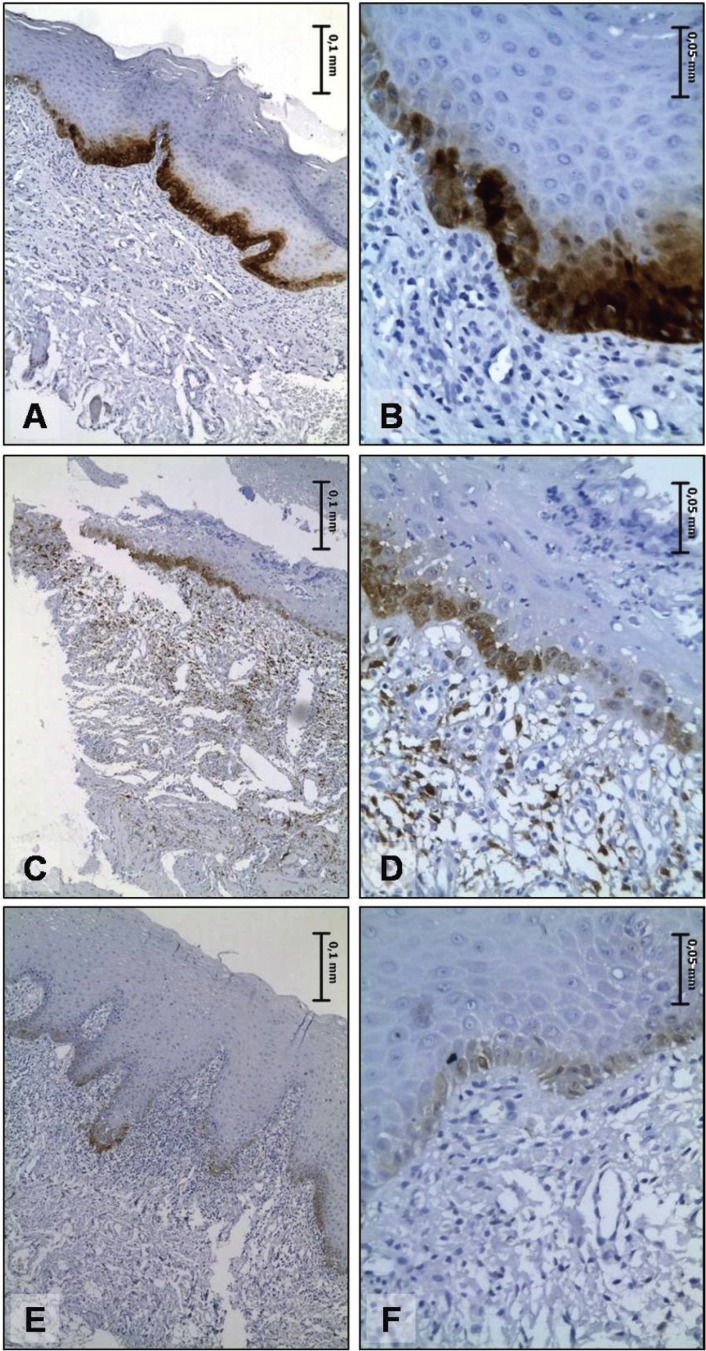
Staining pattern commonly evidenced in the white (A and B) and red (C and D) forms of lichen planus, as well as oral lichenoid lesion (E and F). The MT immunohistochemical reactivity was observed as cytoplasmic and nuclear staining of the cells in the basal layer. This stain was gradually less evident in upper layers.

**Figure 3:**
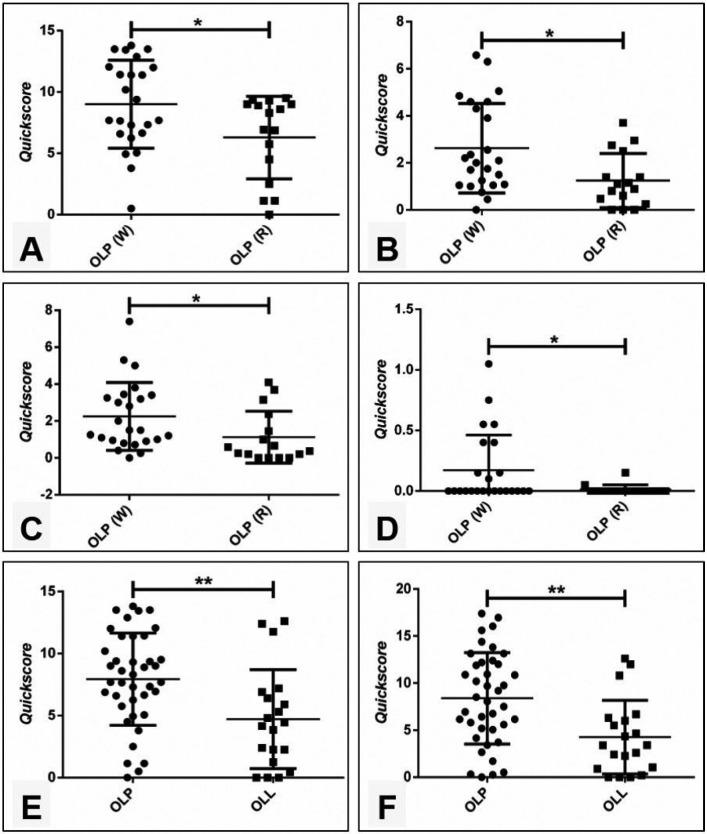
(A) Comparison between the MT staining in the cytoplasmatic compartment of the basal epithelium layer among white and red forms of OLP (Mann-Whitney U test, *p*=0.0467). (B) Comparison between the MT staining in the cytoplasmatic compartment of the parabasal epithelium layer among white and red forms of OLP (Mann-Whitney U test, *p*=0.0159). (C) Comparison between the MT staining in the nuclear compartment of the parabasal epithelium layer among white and red forms of OLP (Mann-Whitney U test, *p*=0.0129). (D) Comparison between the MT staining in the nuclear compartment of the spinous epithelium layer among white and red forms of OLP (Mann-Whitney U test, *p*= 0.0492). (E) Comparison between the MT staining in the cytoplasmatic compartment of the basal epithelium layer among OLP and OLL (Mann-Whitney U test, *p*= 0.0019). (F) Comparison between the MT staining in the nuclear compartment of the basal epithelium layer among OLP and OLL (Mann-Whitney U test, *p*= 0.0011).

The immunohistochemical reactivity for MT was observed as cytoplasmic and nuclear staining of varied intensity, depicting a mosaic pattern. In all groups, staining was more intense in the cells of the basal epithelial layer and was gradually less evident in upper layers. Figure 2 illustrates representative staining patterns for each group. As expected, MT reactivity was more intense in the white forms of OLP when compared to the red ones ( Table 2 and Fig. 3A to 3D). Finally, lesions of OLP depicted significantly higher MT reactivity than OLL ( Table 3 and Fig. 3E and 3F). There was no difference in the MT expression in white OLL and red-white mixed OLL.

**Table 2:**
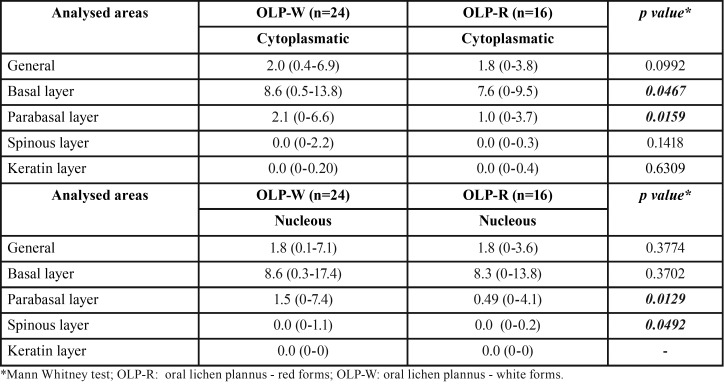
Median Quickscore in white OLP and red OLP groups. Range were presented inside parenthesis.

**Table 3:**
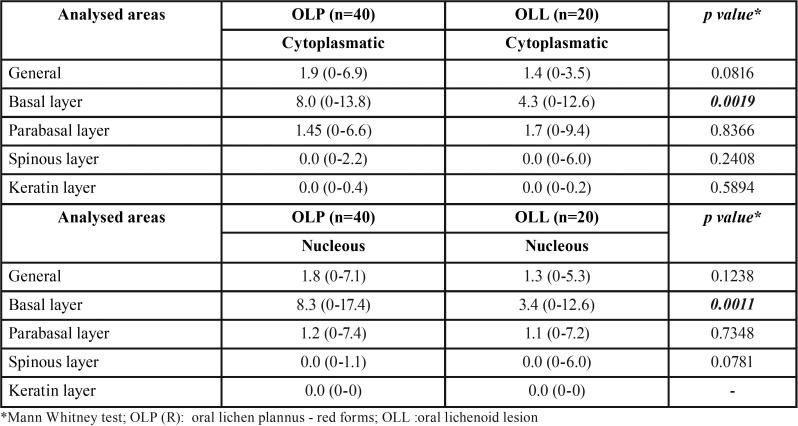
Median Quickscore in OLP and OLR groups. Range were presented inside parenthesis.

## Discussion

In 1978, the World Health Organization (WHO) proposed a clinical and histopathological definition for OLP to facilitate its recognition and differentiation from pre-cancerous disorders (14). This publication has been applied as the golden standard criteria for OLP diagnosis for at least 25 years. However, subsequent studies concluded that this classification has unsatisfactory inter- and intra-observer variability (4,14-18). This inconsistency may be partially explained by the difficulty in separating OLP from several other diseases such as OLL or even lupus erythematosus.

To overcome this problem, Van der Meij and Van der Waal in 2003 proposed a set of revised diagnostic criteria of OLP and OLL, including clinical as well as histopathological aspects (4). This new classification demonstrated less inter- and intra-observer variability and enable pathologist/researchers to perform more reproducible diagnosis, eliminating individual diagnostic variation (4,18). Moreover, such data could lead to better clinical, pathological and etiological characterization of these lesions, dismissing some biases related to the incorrect naming of these disorders. To guarantee more reproducible analysis, we utilized the modified WHO diagnostic criteria of OLP and OLL as proposed in 2003 (4). However, such classification has some limitations, since these proposed criteria are not universally present in all OLP and/or OLL patients.

In the present study, we observed that MT expression is mainly observed in basal and parabasal layers of the oral epithelium, as previously described (11,19,20). In 1992, Hanada and collaborators explained that reactivity for MT in cutaneous lesions of lichen planus was concentrated in the granular layer of the epidermis, in the same way of healthy skin, but was less evident in the former (10). Afterwards, van den Oord and De Ley described the distribution of MT in normal and pathological human skin (21). Although they did not include lichen planus in their analysis, the MT immunohistochemical staining was described as strongly positive in basal keratinocytes of epidermis (21). Interestingly, an MT interrupted staining was found in the basal layer of interface dermatitis (21). In 1998, Matsuura, Tsukifuji, and Shinkai described MT expression in idiopathic annular lichen planus of the skin where MT immune staining increased as well, but it was discontinuous around the rim of erythema and undetectable in the center of the lesion (19). Other authors had depicted MT positivity in lower areas of the epidermis in one case of Palmoplantar lichen planus (20).

Regarding to oral lesions, it was only in 2014 that Allon and co-workers described that epithelial MT expression was significantly higher in keratotic OLP than in atrophic and erosive forms (11). In our work, we also observed that MT expression was less evident in red than white lesions of OLP. These results suggest that MT expression is related to the inflammatory pathogenesis of OLP. In addition, these findings validate our technique, since we were able to reproduce someone else’s data (11).

A previous manuscript has described the MT immunolocalization on amalgam tattoos specimens (12). This paper portrayed that a weak MT positivity was usually seen in inflammatory and endothelial cells close related with amalgam particles (12). The authors affirm the presence of MT immunopositivity in the basal cell layer of the mucosa epithelium in all studied samples (12). Furthermore, MT-staining appears to be stronger in the epithelial basement areas impregnated with powdered amalgam particles (12). Such data reinforces our findings, since we could also evidence a very similar MT immunopositivity in the basal cell layer of OLP and OLL samples. Taken these files together, we could assume that MT expression is always upper-regulated to protect mucosal keratinocytes from variable sources of cellular stress.

Previous studies have classified OLL in four major types based on the method of instigation: oral lichenoid lesions associated with amalgam restorations; oral lichenoid drug reactions; lichenoid lesions in chronic graft-versus-host disease, and lesions that lack an evident triggering agent but have a lichen planus like aspect except for one or more clinical features (1-4,22,23). In these lines, OLL can only be distinguished from OLP by two main features: 1- association with the administration of a drug, contact with a metal (such as dental restorations), foodstuff or systemic disease; and 2- the resolution of the lesions when the causative agent is removed (1-4,22,23). In attempt to facilitate such segregation, authors have suggested that histopathological features such as strict band-like infiltration, atrophic epithelium, saw toothed rete ridges, and Max Joseph space were more frequently seen in OLP than OLL. Conversely, lip involvement, deep connective tissue infiltration, and hyperparakeratosis were described as reliable features for the diagnosis of OLL (1,22).

Several different methods have been proposed to distinguish OLL from OLP (1-4,22-27). Jahanshahi, Ghalayani and Maleki have suggested that the number of degranulated mast cells and greater epithelium thickness were more frequently seen in OLL than in OLP (23). Reddy *et al.* also described an increased number of degranulated mast cells in OLL, when compared with OLP (24). Moreover, they also described an increase in the number of eosinophil and capillaries in OLL than in OLP and normal mucosa (26).

Arreza and collaborators (2014) reported higher expression of COX-2 in OLP when compared with OLL (25). Subsequently, Batu *et al.*, (2016), suggested an increased prolidase activity and oxidative stress and imbalance in the antioxidant defense system in biological fluids of patients with OLP- and OLL-affected patients when compared with the healthy subjects (26). However, OLP and OLL patients revealed almost similar prolidase activity and oxidative stress levels. Rodrigues and collaborators (2017), proposed that the loss of heterozygosity in chromosomes 9p (D9S157, D9S162, D9S171), 11q (D11S1369), and 17p (TP53, AFM238WF2) occurred more frequently in OLL than in OLP (27). Our findings disclosure a weaker MT staining in OLL, when compared to OLP. More studies should be done with larger casuistries to validate these discoveries on a wider scale.

As cited above, many clinical, histopathological, immunological and genetic criteria for differentiating OLP and OLL has been proposed in literature; however, there is currently no established cutoff to facilitate their segregation (1-4,22-27). Furthermore, since most of the described information relies on observational studies, such data must be taken with care because of inherent biases. Since there are few studies involving markers that facilitate the differential diagnosis between the OLP and OLL cases, prospective studies with stronger evidence levels are still needed to better understand the pathogenesis and the trustworthy segregational markers of theses lesions.

In conclusion, the presented results show that differences in MT expression were linked with OLP and OLL etiopathogenesis and clinical appearance. Our data could not support any further clinical implications for the use of MT as a tool in the diagnosis and management of these lesions. Further studies are still necessary to elucidate this topic. In the future, prospective blind controlled trials will answer if MT immune tagging has a real potential to be an OLP diagnosis marker, with prognostic value.
